# Molecular Radiotherapy with ^177^Lu-Immunoliposomes Induces Cytotoxicity in Mesothelioma Cancer Stem Cells In Vitro

**DOI:** 10.3390/ijms23073914

**Published:** 2022-04-01

**Authors:** Tao Huang, Jae Sam Lee, Alexander L. Klibanov, Jiang He

**Affiliations:** 1 Department of Radiology and Medical Imaging, School of Medicine, University of Virginia, Charlottesville, VA 22903, USA; th4g@virginia.edu (T.H.); jsfemto@gmail.com (J.S.L.); 2 Department of Medicine, Division of Cadiovascular Medicine, School of Medicine, University of Virginia, Charlottesville, VA 22903, USA; alk6n@virginia.edu; 3 UVa Cancer Center, School of Medicine, University of Virginia, Charlottesville, VA 22903, USA

**Keywords:** mesothelioma, cancer stem cells (CSCs), molecular radiotherapy, radioimmunotherapy (RIT), liposomes, ^177^Lu

## Abstract

Malignant mesothelioma (MM) is a lethal tumor originating in the mesothelium with high chemotherapeutic resistance. Cancer stem cells (CSCs) persist in tumors and are critical targets responsible for tumor resistance and recurrence. The identification and characterization of CSCs may help develop effective treatment for MM. The objective of this study was to evaluate the therapeutic effect of molecular targeted radiotherapy by ^177^Lu-labeled immunoliposomes (^177^Lu-ILs) on CSCs of mesothelioma. MM CSCs were sorted based on CD26/CD24 expression level and their functional significances were established by small interference RNA. CSC potential of MM was evaluated for drug resistance, cell invasion, and cell growth rate in vitro. CSC metabolism was evaluated with the uptake of ^18^F-FDG. Therapeutic effects of ^177^Lu-labeled immunoliposomes targeting CD26 and CD24 were evaluated in vitro through proliferation and apoptotic assays. CSCs sorted from H28 cells exhibited significant drug resistance and enhanced proliferative activity as well as increased metabolism indicated by higher ^18^F-FDG uptake. Treatment with ^177^Lu-ILs, compared with ^177^Lu-CL and ILs, showed enhanced therapeutic effects on inhibition of proliferation, up-regulation of apoptosis, and suppression of CD26 and CD24 expression. Thus, our results suggest that molecular radiotherapy targeting both CD26 and CD24 could be a promising approach for CSC-targeting therapy for MM.

## 1. Introduction

Malignant mesothelioma (MM) is an aggressive malignance affecting mesothelium and mostly associated with asbestos exposure. After being exposed to asbestos, mesothelioma symptoms can take 20 to 50 years to appear and therefore, the diagnosis is mostly at a late stage [1,2,3]. MM is categorized into three histological subtypes, epithelioid (50–70%), sarcomatoid (10–20%), and biphasic (20–35%) [4,5]. Conventional therapies such as surgery, radiation therapy, and chemotherapy are far from satisfactory, so the prognosis of MM in patients remains poor with median survival of 6–18 months after diagnosis [6,7,8,9]. Epidemiologic studies suggest that due to the long latency period for people already known at risk and the ongoing exposure to asbestos for industrial workers in developing countries, the incidence of MM worldwide is underestimated and will continue to rise [10]. Hence, new approaches to the treatment of MM are desired.

Cancer stem cells (CSCs) have been widely used to explain tumor heterogeneity, resistance to therapy, metastasis, and recurrence [11,12]. It is postulated that tumors are hierarchically organized with a subpopulation of CSCs located at the apex of a pyramid, and these CSCs are responsible for tumor initiation and sustainment through their infinite potential of self-renewal and differentiation into varied progeny forming the bulk of the tumor. Although the origin of CSC remains unclear and controversy arises whether all cancers follow the CSC mode, growing evidence demonstrates that CSCs exist in a wide array of tumors, not only in leukemia [13,14] but also in most solid tumors including breast cancer [15], ovarian cancer [16], pancreatic cancer [17,18], colorectal cancer [19], prostate cancer [20], glioblastoma [21], lung cancer [22], and melanoma [23]. 

More recent studies reported identification and characterization of CSCs in MM and suggest CD26 and CD24 as potential markers and therapeutic targets [24,25]. CD24 is a glycosylated protein highly expressed in numerous cancer and cancer stem cells and involved in the development, invasion, and metastasis of cancer cells [26,27]. Treatment with anti-CD24 mAb significantly inhibited tumor cell growth in a time- and dose-dependent manner by inducing CD24 degradation in vitro and in vivo [28,29]. CD26, known to be involved in metastasis and progression of cancers, is highly expressed in MM, and anti-CD26 mAb inhibited tumor growth in vivo, suggesting that it could be used as a therapeutic target for MM [30,31]. 

Liposomes have been extensively studied and approved as safe materials for drug delivery applications. Nanosized liposomes take advantage of the enhanced permeability and retention (EPR) effect for tumor drug targeting. This makes them versatile carriers of chemotherapy agents for anticancer therapy. Liposomes can be easily tailored to encapsulate therapeutic payloads; their surface can be functionalized with targeting ligands, e.g., antibodies (for immunoliposomes, IL preparation), or peptides. Radiotracers can be applied for simultaneous imaging/detection and therapeutic applications, including combinations of therapy modalities. Particularly, radiolabeled immunoliposomes can be used for specific tumor targeting and provide a high payload of radionuclide per molecule of tumor-targeted antibody. Our lab has been developing immunoliposome-based therapy for MM that has shown specific tumor targeting and may also have potential to combine high payloads of different therapeutic entities including radioisotope and/or chemotherapy agents into the liposome nanoparticles for multimodality therapy [32]. The development of CSC-targeted therapy by radiolabeled immunoliposomes for MM could be more effective and less toxic than the conventional treatment modalities.

In this study, we identified CSCs of MM from several cell lines, namely, H28 (sarcomatoid), H226 (epithelioid), and MSTO-211H (biphasic), based on the expression of CD26 and CD24. The flow cytometry sorting method was used to isolate the subpopulation with higher expression of CD26 and/or CD24 as MM CSCs. Knockdown of these markers by shRNA was employed to evaluate the significance of the marker expressions to CSC signatures such as chemoresistance, proliferation, and invasive potentials in vitro. The uptakes of 2-deoxy-2-[^18^F]fluoro-D-glucose (^18^F-FDG) in glucose-containing and glucose-free media were measured and the results suggested that the metabolism of MM CSCs were more glycolytic than general MM cancer cells. Moreover, therapeutic effects of ^177^Lu-labeled immunoliposomes (^177^Lu-ILs) targeting CD26 and CD24, prepared as previously reported [32], were evaluated in vitro through proliferation and apoptotic assays. The results showed that radioimmunotherapy (RIT) would be a promising CSC-targeted therapeutic modality.

## 2. Results

### 2.1. CD26 and CD24 Are Heterogeneously Expressed on MM Cells

**Figure 1:**
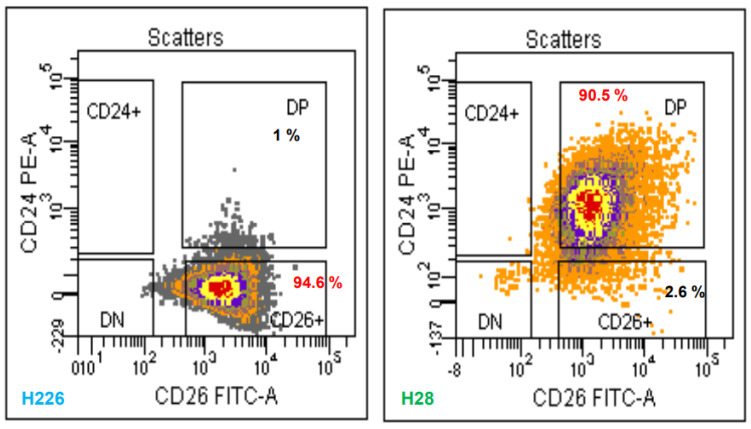
Phenotype expressions of mesothelioma cells, H226, H28, and MSTO-211H. Cells were trypsinized, pelleted, and resuspended in PBS containing 2% FBS. The cells were incubated with CD24-PE, CD26-FITC, and DAPI at 4°C. The double-stained population was sorted by a FACSCalibur flow cytometer.

MM cells representing all subtypes, including H226 (epithelioid), H28 (sarcomatoid), and MSTO-211H (biphasic), were analyzed using a fluorescence-activated cell sorting (FACS) by double staining with anti-CD26 Ab and anti-CD24 Ab (Figure 1). CD26 showed a high surface expression in H226 (>95%) and H28 cells (>93%) but not in MSTO-211H cells (<1%). However, high expression of CD24 was only observed in H28 cells (>90%). A recent study suggested the combination of CD26 and CD24 as CSC markers for MM [25]. Accordingly, we used a highly purified population (~4%) of sorted CD26^high^/CD24^high^ from H28 cells or CD26^high^/CD24^low^ from H226 cells as the tentative CSC source in our study but not the biphasic MSTO-211H cells due to their negative expression of CD26 and CD24.

To assess the functional significance of CD26 and CD24 to MM cells, their protein expressions were inhibited by transducing H28 and H226 cells with short hairpin RNAs (shRNAs) targeting CD26, CD24, CD26/CD24, or non-target control. The expression level was validated after stable cell lines had been established (shCD26, shCD24, and shCD26/24) and sorted by FACS (Table 1). The CD26 expression was reduced by 95% in H28 and 97% in H226 cells, and the CD24 expression was similarly down-regulated by 93% in H28 and 99% in H226 cells. Overall, our results showed that shRNAs caused significant reductions with regard to the expression levels of CD26, CD24, and CD26/24 in H28 and H226 cells compared with the parental or control vector (C-vec) cells.

### 2.2. CSCs Exhibited Significant Resistance to Anti-Cancer Drugs

**Figure 2:**
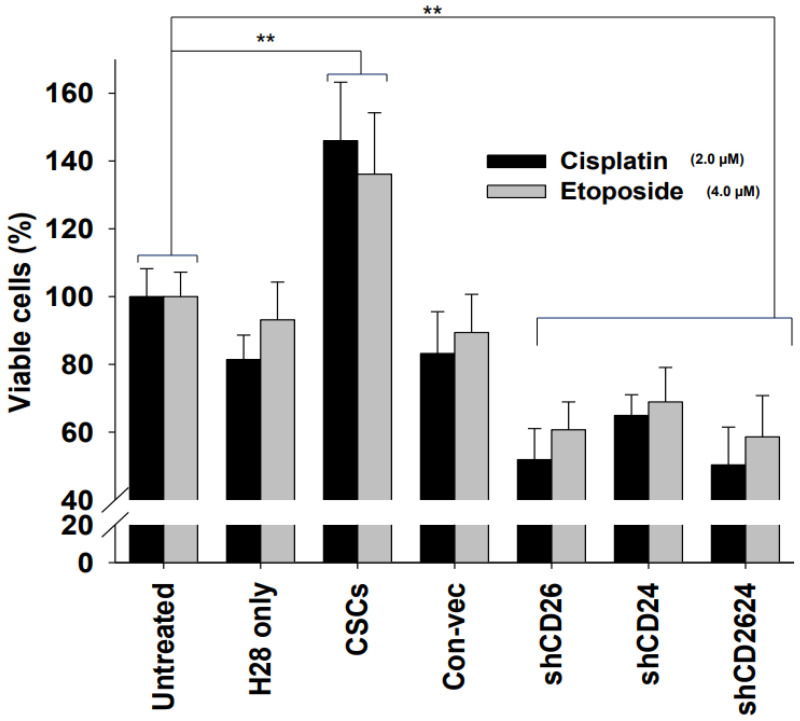
Effects of anti-cancer drugs (cisplatin and etoposide) on cells. MMs were incubated for 24 h and two chemotherapeutic drugs, cisplatin and etoposide, were employed to test drug sensitivity for 1 h. The media were replaced with fresh media. After 72 h, cell viability was measured by CellTiter96 kit. Mean ± SD (*n* = 3), *p* < 0.01 (**).

To investigate the resistance to chemotherapy, sorted CSCs and shRNA cell lines (shCD26, shCD24, and shCD26/24) were exposed to chemotherapeutic drugs (cisplatin and etoposide) for 1 h and their cell toxicities were compared with those of untreated MMs (Figure 2). CSCs exhibited significant resistance to the chemotherapeutic drugs with around 140% cell viability compared with untreated MMs (*p* < 0.001), whereas shRNA cell lines were more sensitive to anti-cancer drugs, resulting in significant cell death. These results indicate that CD26 and CD24 play important roles in drug-resistant activity.

### 2.3. CSCs Increased Invasion Potential and Cell Growth Property

**Figure 3:**
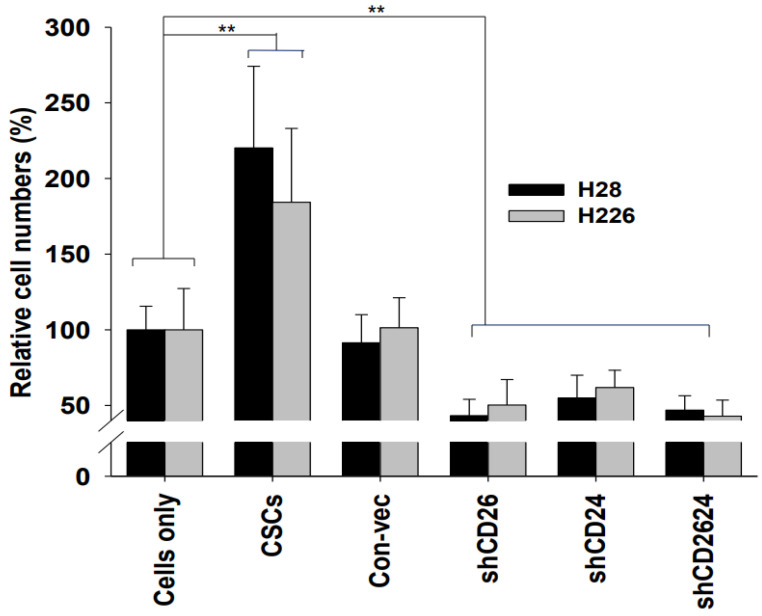
Cell invasion potential of MM cell lines. Cell invasiveness was determined by using a Matrigel invasion chamber. MMs were seeded into the upper chambers in serum-free medium. The outer chambers were filled with medium containing 10% FBS. After 48 h, the non-invading cells were removed by swabbing the top layer. After removal of non-invasive cells, the invaded cells were stained with Diff-Quik Stain Set and quantified. Mean ± SD (*n* = 3), *p* < 0.01 (**).

**Figure 4:**
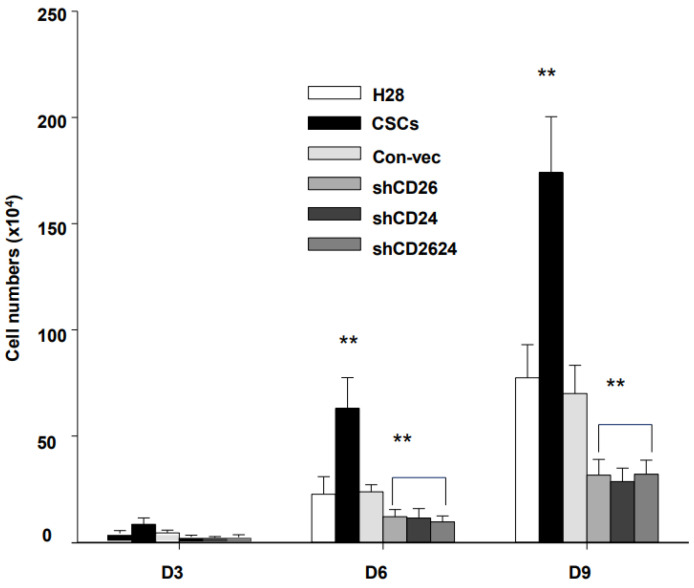
Cell growth assay on MMs of H28. Mean ± SD (*n* = 3), *p* < 0.01 (**).

Invasion assays were performed to compare the invasive potential of sorted CSCs and shRNA cell lines with control MMs (Figure 3). CSCs exhibited the highest invasive activity, while shRNA cells displayed the lowest invasion potential. Efficient down-regulation of CD26 and CD24 led to severely impaired cell invasive property of shRNA cell lines. There was no significant difference in invasive potential between H28 CSCs (CD26^+^24^+^) and H226 CSCs (CD26^+^24^−^). Accordingly, CSC populations of H28 and H226 acquire an enhanced ability to migrate and invade, and these features may account for their highly metastatic behavior in vivo. Their growth property was also compared with untreated MMs to evaluate their proliferation potential. The growth rate of shRNA cell lines was much slower than that of control MMs (Figure 4). On the other hand, CSCs showed extremely enhanced growth rate. Taken together, these data indicate that CD26 and CD24 contribute profoundly to the cell growth and invasion potential of MMs, and the combination of these markers could provide an effective strategy for CSC-targeting immunotherapy.

### 2.4. ^18^F-FDG Uptake In Vitro Was Significantly Increased in CSCs

**Figure 5:**
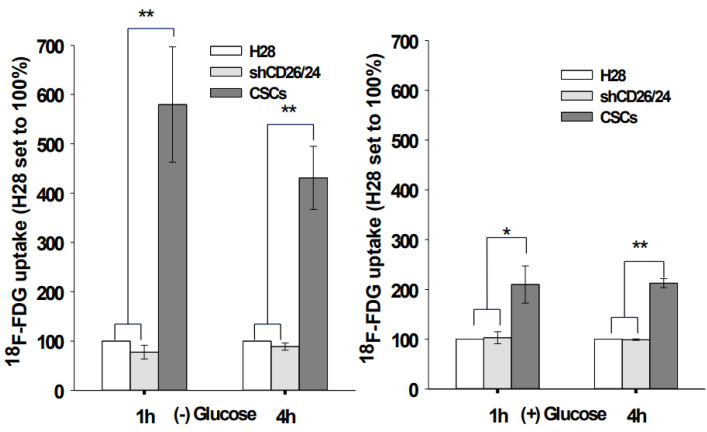
^18^F-FDG uptake in MMs incubated in glucose-free or glucose-containing medium. MMs were incubated with 20 µCi of ^18^F-FDG for 1 or 4 h at 37 °C. In some plates, glucose was excluded from media because it competes with FDG uptake. After removal of excess tracer, cellular tracer uptake was measured by using a gamma counter to evaluate metabolic activity in MMs and tracer uptake was corrected for the number of cells. ^18^F-FDG uptake in shRNA and CSCs was compared with that in H28 cells. Mean ± SD (*n* = 3), *p* < 0.05 (*), and *p* < 0.01 (**).

2-deoxy-2-[^18^F]fluoro-D-glucose (^18^F-FDG) is a commonly used radiotracer for positron emission tomography (PET) in oncology. Its use helps detect metabolic differences between normal and tumor tissues. Accordingly, ^18^F-FDG uptake of CSCs (CD26^+^24^+^ of H28) and shRNA cells (shCD26/24 of H28) was analyzed and compared with that of control H28 cells to evaluate their metabolic activities. ^18^F-FDG uptake of CSCs was significantly increased up to 600% in the glucose depleted media and 200% in the glucose-containing media (Figure 5) compared to that of H28 cells. As expected, the ^18^F-FDG uptake of CSCs in the glucose-containing media was decreased because of competition. During a period of 4 h, the ^18^F-FDG uptake of CSCs in the glucose-free media was decreased from 580% to 430% while that in the glucose-containing media was sustained. There was no significant ^18^F-FDG uptake difference between H28 and shRNA cells in both culture conditions. Our data indicate that CSCs have a high metabolic activity in a glycolytic manner.

### 2.5. Preparation and Radiolabeling of CLs and ILs

Liposomes were prepared by thin lipid film hydration followed by sonication and extrusion as previously reported [32]. For the preparation of ILs, the cysteine residues in heavy chain of CD26 (202.36) or CD24 (SN3) were reduced for conjugation with DSPE-PEG_2000_-MAL to obtain DSPE-PEG_2000_-CD26 and DSPE-PEG_2000_-CD24. The efficiency of cysteine reduction was >85% as measured by Ellman’s reagent assay. 

**Figure 6:**
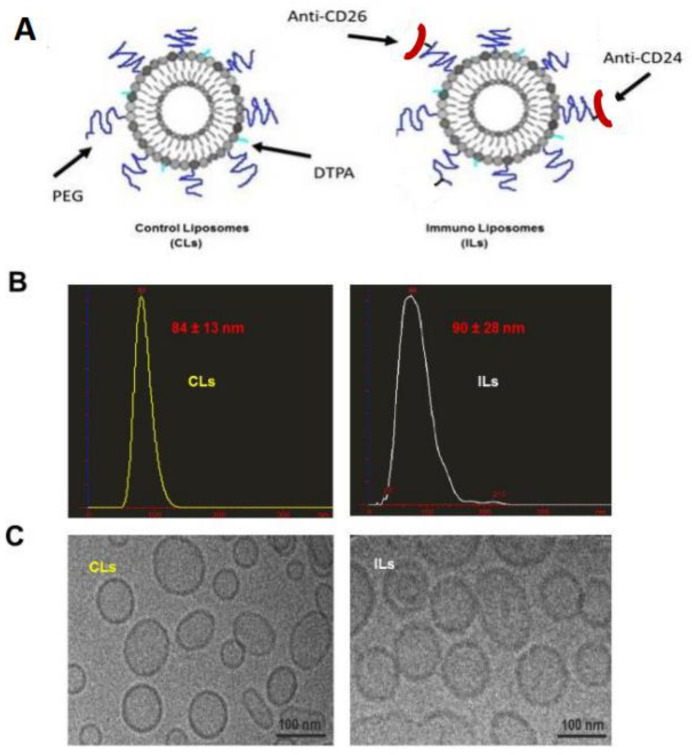
Formulation and characterization of CLs and ILs. (**A**) To obtain ILs, the reduced mABs (CD26 and CD24) were conjugated to Mal-PEG-DSPE first and the resultant conjugates, DSPE-PEG_2000_-CD26 and DSPE-PEG_2000_-CD24, were inserted onto liposomes through a post-insertion method as described in the Materials and Methods section. (**B**) The size distribution of CLs and ILs were measured by LM10 nanoparticle tracking system. (**C**) Cryo-electron micrograph of CLs and ILs. Particles are predominantly spherical and consist of subpopulations with different diameters. Scale bar: 100 nm.

The modified antibodies (DSPE-PEG_2000_-CD26 and DSPE-PEG_2000_-CD24) were incorporated onto liposomes via a post-insertion method [32]. The BCA protein assay was performed to assess antibody conjugation onto liposomes; typically, 75–82% of the added antibodies were coupled. The particle sizes of CLs and ILs were measured by a nanoparticle tracking system (NTS) to be 84 ± 13 nm and 90 ± 28 nm, respectively (Figure 6B). The size and morphology of CLs and ILs were further characterized using a cryogenic transmission electron microscope (CryoTEM) (Figure 6C). It showed that CLs and ILs had quite spherical and uniform size with an average size of 85–95 nm. The size of liposomes remained unchanged upon storage at 4 °C for one month.

To facilitate radiolabeling of CLs and ILs, a DTPA-conjugated phospholipid (DMPE-DTPA) was incorporated in the liposome preparation as reported previously [32]. The success of radiolabeling was confirmed by size exclusion chromatography and the radiolabeling efficiency was >95% in both ^177^Lu-CLs and ^177^Lu-ILs as determined by recovery from the PD-10 column. The in vitro stability was tested in PBS and 10% serum at 37 °C for up to 36 h, and no obvious decomposition was observed as we previously reported [32].

### 2.6. RIT (^177^Lu-ILs) Inhibited Proliferation of MMs by Activating Apoptotic Activity In Vitro

**Figure 7:**
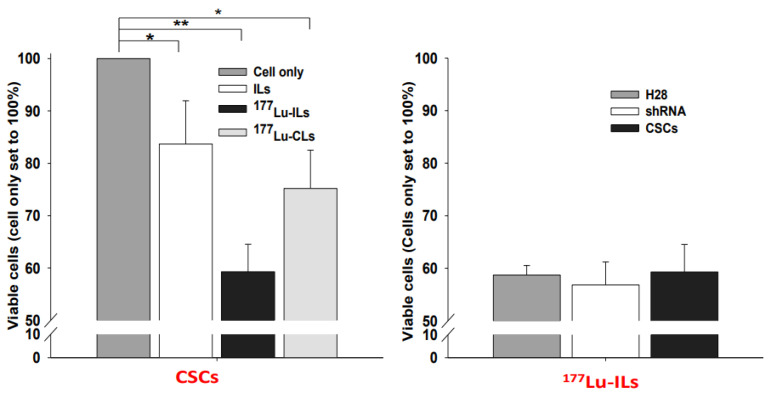
Cytotoxicity in CSCs treated with ^177^Lu-ILs or ^177^Lu-CLs. MMs were plated and incubated overnight to allow cell attachment. The MMs were treated with ^177^Lu-IL or ^177^Lu-CL. Control wells were treated with media alone or ILs only. After 1 h incubation, the media were replaced with fresh media, further incubated for 48 h, and cell viability was determined using MTS. Percentage survival was calculated by setting untreated CSCs as 100% (left). MM cell lines treated with ^177^Lu-ILs were compared with untreated cells (right). Mean ± SD (*n* = 3), *p* < 0.05 (*), and *p* < 0.01 (**).

The therapeutic effects of ILs, ^177^Lu-CLs, and ^177^Lu-ILs on the CSCs, shRNA cells, and H28 cells were determined by an MTS assay, and their cell viabilities were compared with an untreated control (Figure 7). First, the effect of each therapeutic agent was evaluated in CSCs (Figure 7, left panel). Significant reductions in cell proliferation were detected in all treatment groups compared with untreated cells. ^177^Lu-ILs (*p* < 0.01) induced significant cytotoxicity on CSCs and H28 cells as well as shRNA cells (Figure 7, right panel). Over 40% of CSCs’ viability was reduced by ^177^Lu-ILs treatment compared with CSC itself. There was no significant cytotoxic difference between ILs and ^177^Lu-CLs to the MMs. Thus, immunotherapy by anti-CD26/anti-CD24 antibody combination in itself is moderately effective, same as the non-targeted radiotherapy (^177^Lu-CLs). Next, the effect of individual therapeutic agent was evaluated in MMs. 

**Figure 8:**
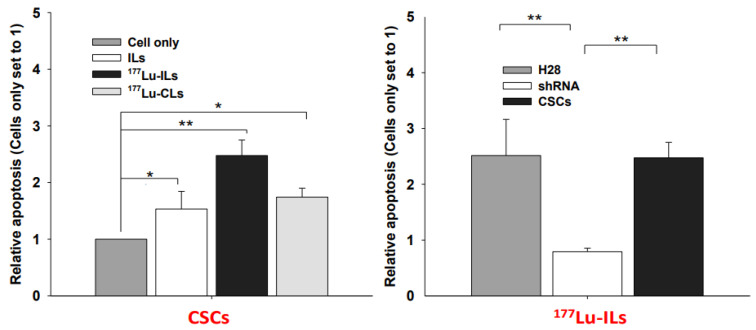
Induced apoptosis in MM cells treated with ^177^Lu-ILs or ^177^Lu-CLs. Left panel: MM CSCs were treated with ^177^Lu-IL or ^177^Lu-CL for 1 h. Control was treated with media alone or ILs only. The media were replaced with fresh media and further incubated for 48 h. Apoptosis was assessed using a Cell Death Detection ELISA^PLUS^ and calculated by setting untreated CSCs as 1. Right panel: MM cells treated with ^177^Lu-ILs were compared with untreated cells. Mean ± SD (*n* = 3), *p* < 0.05 (*), and *p* < 0.01 (**).

Apoptosis of MMs induced by ILs, ^177^Lu-CLs, and ^177^Lu-ILs was also performed in vitro (Figure 8). As shown in the Figure 8 (left panel), significantly increased apoptosis of CSCs was detected in all treatment groups compared with untreated cells. ^177^Lu-ILs (*p* < 0.01) induced significant apoptosis of CSCs and H28 cells, but not shRNA cells, presumably due to reduced targets of CD24 and 26 (Figure 8, right panel). ^177^Lu-ILs treatment induced the most significant apoptosis of CSCs compared to ILs and ^177^Lu-CLs, which may be attributed to double effects from immunotherapy from two antibodies and the radiation from ^177^Lu. Taken together, our results suggest that RIT is a promising therapeutic modality for targeting CSCs due to its enhanced tumor cell killing ability.

## 3. Discussion 

CSCs are a subset of cancer cells that possess the capacity to self-renew and initiate tumor growth. Analogue to normal stem cells, the defining characteristics of CSCs lie in their extensive proliferative potential and their ability to generate phenotypically and biochemically diverse progeny tumor cells [11]. CSC are involved in cancer metastasis, in resistance to therapeutic treatment, and in the relapse after initial response to therapies [33]. According to CSC hypothesis, eradication of CSCs can make cancers curable. Therefore, CSC-targeting therapy holds great potential for cancer treatment.

In this study, we evaluated phenotype expressions of CD26 and CD24 in three typical MM cell lines, H28 (sarcomatoid), H226 (epithelioid), and MSTO-211H (biphasic), through flow cytometry. Our results showed that CD26 was highly expressed on H28 and H226 cells while CD24 had high expression only on H28 cells. The expression of CD26 was closely correlated with that of CD24 in sarcomatoid-type cell lines (H28), but no similar pattern was observed from the other two cell lines. This result was consistent with the previous report, which showed that these CSC markers may differ depending on the histological subtypes [25]. Moreover, knockdown of CD26 and/or CD24 expression was achieved by shRNAs in both H28 and H266 cell lines. The properties of CD26CD24-enriched CSCs, CD26CD24-depleted cells (shCD26CD24), and parent cells/control cells (Con-vec) were characterized with regard to growth activity, drug resistance, and invasive potential. The CSCs showed significantly increased growing activity, resistance to chemotherapy, and invasive potential than parent cells, whereas CD26CD24-deficient cell lines displayed slower growth, more sensitivity to drug treatment, and less invasion (*p* < 0.001). These results confirmed the potential of CD26 and CD24 as MM CSC markers and can be valuable targets for MM treatment.

High uptake of glucose by tumor cells constitutes the biochemical basis for cancer imaging with ^18^F-FDG PET technology [33]. In our study, we estimated the glucose uptake of MM CSCs by incubating with ^18^F-FDG, a glucose analog which is preferentially taken up into cells with high glycolytic metabolism and becomes metabolically trapped in the cell. By measuring the radioactivity within the cell, we can compare the glucose uptake of MM CSCs with the non-CSCs. Our results demonstrate that the ^18^F-FDG uptake of CSCs was significantly increased, up to 600% in the glucose-depleted media (Figure 5 left) and 200% in the glucose-containing media (Figure 5 right), suggesting that the MM CSCs have higher glycolytic activity than the differentiated MM cancer cells. The finding that CSCs are highly dependent on glycolysis is consistent with the Warburg hypothesis, which attributes the origin of cancer to mitochondrial respiration injury and hence a switch from mitochondrial oxidative phosphorylation to cytosolic glycolysis [34]. CSCs may be metabolically heterogeneous to offer survival advantages under various microenvironments, or they can switch from mitochondrial respiration to glycolytic metabolism under certain conditions.

Therapeutic effects of multiple modalities on CSCs were evaluated in vitro. Based on MTS assay of cell viabilities and proliferation assay of apoptosis, immunotherapy (ILs) and radiotherapy (^177^Lu-CLs) showed comparable efficacy with each other while RIT (^177^Lu-ILs) was more efficient in killing CSCs than the other two modalities (Figure 7 left and Figure 8 left). This therapeutic effect may be attributed to RIT that possessed both capabilities of immunotherapy (anti-CD26, anti-CD24) and radiotherapy (^177^Lu). No noticeable difference was observed in the treatment results of ^177^Lu-ILs on CSCs, H28, or shRNA cells (Figure 7 right), probably due to overdoses that flattened the difference. However, ^177^Lu-ILs did induce more apoptosis in CSCs and H28 cells than in shRNA cells (target-deficient control cells) (Figure 8 right) that may be attributed to knockdown of targets of CD24 and CD26 in shRNA cells.

Although many markers have been proposed to identify and isolate CSCs in many solid tumors, none of them are universal to various tumors or specific to certain cancer types. A combination of two or multiple markers may facilitate the identification and isolation of CSCs, but many observations and implications remain ambiguous and yet to be concluded. For example, our data as well as other results [24,25] showed heterogeneous expression of CSC markers among MM subtypes, particularly for CD24, which is considered a common CSC marker in various tumors. Expression of CSC markers varies greatly between cell lines, even for the cells of the same cancer subtype. Despite accumulating evidence from numerous studies, questions regarding heterogeneity between and amongst cancer subtypes and functional properties of CSC markers are still far from full elucidation.

Moreover, CSCs have adaptive behavior in the heterogeneous microenvironment such as hypoxia, nutrition, and low pH [35,36,37], which may play an important role in CSC progression. Since in vitro studies cannot completely mimic the specific tumor microenvironment, the characterization of CSCs from sorted and cultured cell populations may reflect the altered status which is different from the authentic CSC state in tumor. One study suggested CD26 status may be altered and thus display diverse effects on the growth and metastatic potential in various tumors, explaining the discrepancy in the observation that the absence of CD26 is associated with the development of some cancers, whereas the presence of CD26 is related to a more aggressive phenotype in other tumors [38]. More investigations are necessary to elucidate the mechanisms or pathways and confirm the enhanced therapeutic effects of the combination of immunotherapy targeting CSCs and radiation therapy.

In summary, the three MM cell lines (H226, H28, and MSTO-211H) showed heterogeneous expression of CD26 and CD24. The CD26^+^/CD24^+^-enriched cells from sarcomatoid H28 cell lines and the CD26^+^/CD24^−^-enriched cells from epithelioid H226 cell lines showed higher resistance to chemotherapy and higher invasion potential, whereas MM cells with CD26 and CD24 knockdown were more responsive to chemotherapy, suggesting that CD26 and CD24 are potential CSC markers for certain histological subtypes of MM. The ^18^F-FDG uptake experiment demonstrated that MM CSCs based on CD26^+^/CD24^+^ undergo more active metabolism through glycolysis. The initial RIT results in vitro showed the potential of CD26 and CD24 as targets for CSC-oriented therapy. Further in vivo study is warranted to validate the RIT therapeutic efficacy. 

## 4. Materials and Methods 

### 4.1. Reagents and Materials

All the lipids and their derivatives, including 1-hexadecanoyl-2-(9Z-octadecenoyl)-*sn*-glycero-3-phosphocholine (POPC), 1,2dimyristoyl-*sn*-glycero-3-phospho-ethanolamine-N-diethylene-triamine-pentaaceticacid (DMPE-DTPA), 1,2-distearoyl-*sn*-glycero-3-phosphoethanolamine-N-[amino(polyethyleneglycol)-2000] (DSPE-mPEG_2000_), 1,2-distearoyl-*sn*-glycero-3-phospho-ethanolamine-N-[maleimide(polyethyleneglycol)-2000] (DSPE-PEG_2000_-MAL), and cholesterol, as well as the mini extruder were purchased from Avanti Polar Lipids (Alabaster, AL, USA). 2-mercaptoethanolamine (2-MEA) was purchased from Thermo Scientific (Wilmington, DE). Dimethyl sulfoxide (DMSO, anhydrous), chloroform (anhydrous), and phosphate-buffered saline (PBS) were purchased from Sigma-Aldrich (St. Louis, MO, USA). PD-10 columns (Sephadex G-25) were purchased from GE healthcare (Chicago, IL, USA). ^177^LuCl_3_ was purchased from Perkin Elmer (Boston, MA, USA).

### 4.2. Cells 

Human malignant mesothelioma cell lines, NCI-H226 (Epithelioid), NCI-H28 (Sarcomatoid), and MSTO-211H (Biphasic), were purchased from American Type Culture Collection (Manassas, VA, USA). Cells were incubated in culture medium, consisting of RPMI 1640 supplemented with 10% FBS, penicillin (100 units/mL), and streptomycin (100 μg/mL) (GIBCO BRL, Rockville, MD, USA) at 37 °C in a humidified atmosphere of 5% CO_2_. CD24 and CD26 expression of mesothelioma cell lines was evaluated by flow cytometry. Briefly, cells were trypsinized, pelleted, and re-suspended in PBS containing 2% FBS. The cells (1 × 10^5^ cells in 500 µL) were incubated with CD24-PE, CD26-FITC, and DAPI (BD-Pharmingen, San Jose, CA, USA) for 1 h at 4 °C and washed three times with PBS containing 2% FBS. The purity of sorted cells was analyzed by a FACSCalibur flow cytometer with CellQuest software (BD, San Jose, CA, USA). 

### 4.3. shRNA Lentiviral Transduction

To assess the functional significance of CD24 and CD26 in mesothelioma cells, the expression of CD24 and CD26 was down-regulated using shRNA lentiviral particles (MISSION; Sigma-Aldrich, St. Louis, MO, USA). Lentiviral transduction of cells with particles for shRNAs targeting CD26 (SHCLNV-NM_001935), CD24 (SHCLNV-NM_013230), or non-target control (SHC001V) was performed based on the manufacturer’s protocol. Briefly, 1 × 10^5^ of NCI-H226 or NCI-H28 cells in RPMI 1640 medium were plated in 12-well plates. After 24 h, hexadimethrine bromide (Sigma-Aldrich), a transduction enhancer, was added to each well (8 μg/mL); 2 × 10^5^ of viral particles (control vector, shCD26, shCD24, or mixture of shCD26/shCD24) were added to each well; and the cell-viral particle mixture was incubated overnight at 37 °C. After overnight incubation, the medium containing viral particles was removed and replaced with fresh medium containing puromycin (2 μg/mL) for the selection of transduced cells. Before lentiviral transduction, a puromycin titration was performed to identify the minimum concentration of puromycin causing complete cell death. Cells grown to ∼90% confluency were trypsin treated and sub-cultured in fresh medium containing puromycin. Stable cell lines were collected at day 14, analyzed by flow cytometry, and used for in vitro assays (anti-cancer drug assay, invasion assay, and growth assay). The shRNA target sequences are as follows:

CD24: ccggcttctgcatctctactcttaactcgagttaagagtagagatgcagaagtttttg (NM_013230.2-333s21c1).

CD26: ccgggactgaagttatactccttaactcgagttaaggagtataacttcagtctttttg (NM_01935.2-271s1c1). 

### 4.4. Drug Sensitivity Assay 

To examine the anti-cancer drug effects in MMs (H28/H226, shRNA transduced, or CSCs), 2 × 10^3^ of MMs per well were seeded on 96-well plates for 24 h, and then two chemotherapeutic drugs, cisplatin (2.0 µM) and etoposide (4.0 µM), were employed to test the drug sensitivity for 1 h. The media were replaced with 100 μL of fresh media. After 72 h incubation, cell viability was checked by CellTiter96 kit (Promega, Madison, WI, USA). At the time of assay, 20 μL of MTS reagent was added to control and treatment wells and the plates were incubated for another 4 h at 37°C. The absorbance of formazan reduced from MTS by metabolically active cells was measured at 490 nm using a SpectraMax 190 microplate reader (Molecular Devices, Sunnyvale, CA, USA). The absolute absorbance for each group was determined by subtracting the background absorbance. All assays were performed in triplicate and the results are expressed as a mean ± SD.

### 4.5. Cell Invasion and Growth Rate Assay 

Cell invasiveness was determined by using a Matrigel invasion chamber according to the manufacturer’s instructions (BD Biosciences, San Jose, CA, USA). Briefly, 2.5 × 10^4^ MM cells (H28/H226, shRNA transduced, or CSCs) were seeded into the upper chambers in serum-free RPMI1640. The outer chambers were filled with RPMI1640 containing 10% FBS. After 48 h incubation, the non-invading cells were removed by swabbing the top layer. After removal of non-invasive cells, the invaded cells were stained with Diff-Quik Stain Set (Siemens, Newark, DE, USA) and quantified. For growth rate analysis, 1 × 10^4^ MM cells (H28/H226, shRNA transduced, or CSCs) were seeded on 24-well plates and serial passages were established in 12- or 6-well plates for up to 9 days. The total cell numbers were counted using a Zeiss Axiovert 200 microscope (Thornwood, NY, USA).

### 4.6. In Vitro ^18^F-FDG Uptake

NCI-H28 sarcomatoid MM cells (H28/H226, shRNA transduced, or CSCs) were plated in six 24-well plates at 7.5 × 10^4^/500 µL. After 24 h incubation, RPMI 1640 culture media were replaced with fresh media containing 20 µCi (0.75 MBq) of ^18^F-FDG (PETNET Solutions, Inc., Charlottesville, VA, USA) and incubated for 1 or 4 h at 37 °C. In some plates, glucose was excluded from RPMI 1640 medium because it competes with FDG uptake. After removal of excess tracer, cellular tracer uptake was measured by using a 2480 Wizard^2^ gamma counter (PerkinElmer, Waltham, MA, USA) to evaluate metabolic activity in MMs, and tracer uptake was corrected for the number of cells.

### 4.7. Preparation of Liposomes

Liposomes were prepared by thin lipid film hydration followed by sonication and extrusion [28,29]. Briefly, liposomes composed of POPC, cholesterol, DSPE-mPEG_2000_, and DMPE-DTPA were combined in a molar ratio of 200:133:0.1:0.03, respectively. A thin lipid film was formed by evaporating the solvent on a rotary evaporator under vacuum in a 10 mL round bottom flask. The lipid layer was further dried overnight in a vacuum desiccator. Each liposome batch consisted of 18 μmol phospholipid and was rehydrated in 3 mL of PBS (pH 7.4). Hydration of the lipid films was performed at room temperature (RT), involving vigorous vortexing for 15 min, followed by sonication (Aquasonic 75T, VWR, Radnor, PA, USA) for 30 min. The resulting multi-lamellar liposomes were repeatedly extruded (21 times) at RT through Whatman^®^ polycarbonate membrane filters (Cytiva Life Science, Marlborough, MA, USA) by gradually decreasing pore size of 0.2, 0.1, and 0.08 μm, using a mini-extruder (Avanti Polar Lipids Inc.), yielding unilamellar liposomes of ~100 nm diameter. Hydrodynamic diameter of the liposomes was determined by Submicron Particle Sizer (Nicomp 370 DLS, Particle Sizing Systems, Inc., Santa Barbara, CA, USA). The phospholipid concentration of the liposomes was determined by a phosphorous assay.

### 4.8. Preparation of Immunoliposomes (ILs)

The reduction of cysteine residues in the single-chain variable fragments of CD26 (202.36) and CD24 (SN3, Santa Cruz Biotechnology, INC., Dallas, TX, USA) was performed according to the manufacturer’s instructions (Thermo Scientific, Wilmington, DE, USA). Briefly, 200 µg/mL of CD26 or CD24 was mixed with 6 mg of 2-MEA (final 50 mM) adjusted with 0.5 M EDTA (pH 8.5) for 1.5 h at 37 °C. The efficiency of cysteine reduction was assayed using Ellman’s reagent (Thermo Scientific, Wilmington, DE, USA). Subsequently, the reduced CD26 or CD24 was purified using PD-10 column (GE Healthcare, Piscataway, NJ, USA) equilibrated with 0.1 M NaHCO_3_/1 mM EDTA buffer (pH 8.0). The purified fraction was concentrated using the Amicon^®^ Ultra-4 centrifugal filtration device (Millipore, Milford, MA, USA) with a 10 kDa MWCO filter at 4000 rpm for 15 min. The final concentration of CD26 or CD24 was determined by both a NanoDrop UV spectrometer and a Micro BCA Protein Assay Kit (Thermo Scientific, Wilmington, DE, USA).

The reduced CD26 or CD24 antibodies were conjugated to DSPE-PEG_2000_-Mal. Briefly, 5 μL DSPE-PEG_2000_-Mal stock solution (20 mg/mL in dry DMSO) was reacted with 100 μL (200 μg) of reduced CD26 or CD24 antibodies in 0.1 M NaHCO_3_/1 mM EDTA buffer (pH 8.0) for 1.5 h under RT. Subsequently, the conjugate was purified by PD-10 column with PBS (pH 7.4). Purified fractions were pooled and concentrated by using an Ultra-4 centrifugal filtration device with a 10 kDa MWCO filter. 

To obtain ILs, DSPE-PEG_2000_-CD26 and DSPE-PEG_2000_-CD24 were incorporated onto liposomes through a post-insertion method [32]. In brief, 2 mM (200 µL) of liposomes was incubated with 5 µg of each DSPE-PEG_2000_-CD26 and DSPE-PEG_2000_-CD24 for 1 h under mild rotation. The purification of mAb-anchored ILs was performed using a PD-10 column with PBS as a mobile phase.

### 4.9. Characterization of Control Liposomes (CLs) and ILs

Hydrodynamic diameters of CLs and ILs were measured using an LM10 nanoparticle tracking system equipped with a 640 nm laser in a range of concentration 1–10 × 10^8^ nanoparticles per ml at 23 °C and calculated with NTA 2.0 software (NanoSight Ltd., Mebane, NC, USA). For cryogenic electron microscopy (cryoEM) observation of CLs and ILs, 2 μL aliquots of each preparation were applied to freshly glow-discharged, holey carbon grids, blotted to near dryness, and flash frozen in slurry of liquid ethane. Grids were maintained at liquid nitrogen temperatures in a Gatan 626 cryo-holder and examined using the FEI Tecnai G^2^ Spirit BioTwin with tomography (Hillsboro, OR, USA) under low electron dose conditions. Micrographs with minimal astigmatism and drift were digitized on a Zeiss microdensitometer (Z/I Imaging) at a 7 μm sampling interval corresponding to 2.0 or 1.56 Å resolution at the specimen. 

### 4.10. Radiolabeling of CLs and ILs with ^177^Lu

For the purpose of radiolabeling the liposomes, 4 mCi of ^177^Lu was typically used. A total of 20 μL (0.05 N HCl) of ^177^LuCl_3_ (Perkin Elmer Inc.,) was taken in a tube and 9 μL of 0.5 M sodium acetate was added to adjust the pH to ~5. Then, 100 μL (200 µg) of ILs or non-targeted CLs was added to the tubes and incubated for 1 h at RT (~20 µCi/µg ILs). Subsequently, the ^177^Lu-labeled CLs (^177^Lu-CL) or ILs (^177^Lu-IL) were purified by using a PD-10 column with PBS as a mobile phase. Labeling efficiency of ^177^Lu-CL or ^177^Lu-IL was calculated from the ratio of radioactivity associated with the liposome fraction compared to the total added radioactivity. The radioactivity was measured with the CRC-55tW Dose Calibrator/Well Counter (Capintec, Inc., Ramsey, NJ, USA).

### 4.11. In Vitro Cell Proliferation Assay Treated with ^177^Lu-IL or ^177^Lu-CL

Cells (H28, shRNA transduced, or CSCs) suspended in RPMI-1640 media were plated at a density of 5.0 × 10^3^ cells/well in 96-well plates and then incubated in a humidified incubator at 37 °C with 5% CO_2_ overnight to allow cell attachment. The cells were treated with ^177^Lu-IL or ^177^Lu-CL at 30 µCi (1.5 µg ILs)/well. Control wells were treated with media alone and ILs in ~1.5 µg/well. After incubation of 1 h, the media were replaced with 100 μL of fresh media, further incubated for 48 h, and cell viability was determined using MTS in the colorimetric proliferation assay according to the manufacturer’s protocol.

### 4.12. In Vitro Cell Death Assay Induced by ^177^Lu-IL or ^177^Lu-CL

Cell death, i.e., apoptosis or necrosis, resulting from treatment with ^177^Lu-IL or ^177^Lu-CL was assessed using the Cell Death Detection ELISA^PLUS^ (Roche Applied Science, Indianapolis, IN, USA) according to the manufacturer’s protocol. Briefly, 5.0 × 10^3^ cells MMs (H28, shRNA transduced, CSCs) were seeded on 96-well plates and incubated at 37 °C with 5% CO_2_ overnight to allow cell attachment. The cells were treated with ^177^Lu-IL or ^177^Lu-CL at 30 µCi (1.5 µg ILs)/well for 1 h. Control wells were treated with media alone or ILs in ~1.5 µg/well. The media were replaced with 100 μL of fresh media and further incubated for 48 h. The plate was centrifuged at 200 g for 10 min. The supernatant layer containing the necrotic fraction was transferred into a glass vial and stored at 4 °C until further analysis for necrosis. The cell pellet containing apoptotic bodies was resuspended in a lysis buffer and incubated for 30 min at RT. The plate was centrifuged (200 g for 10 min) and supernatant solutions containing the necrotic fraction and the cell lysate solutions containing the apoptotic fraction (20 μL) were placed in triplicate into the wells of a streptavidin-coated microplate, to which we added 80 μL of the reagent containing a mixture of anti-histone–biotin and anti-DNA–peroxidase. The plate was covered with an adhesive cover foil and incubated at 20 °C for 2 h in a shaking incubator at 250 rpm. During the incubation period, the anti-histone antibody binds to the histone component of the nucleosomes and simultaneously captures the immunocomplex to the streptavidin-coated microplate via its biotinylation. At the same time, the anti-DNA–peroxidase antibody reacts with the DNA component of nucleosomes. Unbound antibodies were thoroughly washed by incubation buffer. The amount of nucleosomes retained by the peroxidase in the immunocomplex, corresponding to the extent of apoptosis and necrosis, was quantitatively determined photometrically with ABTS (2,2′-azinobis-3-ethyl-benzothiazoline-6-sulfonic acid) as a substrate by using a SpectraMax 190 microplate reader at a wavelength 405 nm and a reference wavelength of 490 nm.

### 4.13. Statistical Analysis

All data are reported as mean ± SD. Statistical analysis was performed using an IBM SPSS Statistics. Student’s t-test and ANOVA were used to compare results. Data were considered as statistically significant for *p* < 0.05.

## Figures and Tables

**Table 1:** Expression of CD24 and CD26 was down-regulated in MMs using shRNA lentiviral particles. The results are based on flow cytometry analysis.

H28	CD26^+^	CD24^+^	H226	CD26^+^	CD24^+^
C-vec	81.5%	71.3%	C-vec	76.1%	1.09%
sh-CD26	4.47%	-	sh-CD26	2.12%	-
sh-CD24	-	6.1%	sh-CD24	-	0.6%
sh-CD26/24	4,75%	6.52%	sh-CD26/24	4.19%	3.18%
	CD26^−^24^−^ = 92.1%			CD26^−^24^−^ = 94.39%	

## Data Availability

The datasets used and/or analyzed during the current study are available from the corresponding author on reasonable request.

## Abbreviations

MM: malignant mesothelioma; CSC: cancer stem cells; RIT: radioimmunotherapy; PET: positron emission tomography; ^177^Lu-ILs: ^177^Lu-labeled immunoliposomes; ^177^Lu-CLs: ^177^Lu-labeled control liposomes with no antibody conjugates; shRNA: short hairpin RNA; mAB: monoclonal antibody.
